# Case of Hydrophobia

**Published:** 1889-09

**Authors:** J. D. Bradley

**Affiliations:** Kingston, Ga.


CASE OF HYROPHOBIA.—We give place to the following letter, addressed to Dr. Gaston, of this City:

Kingston, Ga., Aug. 14th, 1889.

PROF. J. MCF. GASTON,

DEAR DOCTOR:—

I would like to give you synopsis of a case coming under my observation. Norman Doyle, a negro boy 15 years old, came to me June 20th, to dress his arm, which had been bitten on the wrist by a dog. It was a suspicious dog he met in the road. The boy struck the dog with a stick before he was bitten. The dog made his escape and it is unknown what became of the dog.

Two wounds were made on the arm, but were insignificant. I made an ordinary dressing with iodoform, believing there were no cases of rabies in North Georgia. My ideas were in accordance with Youatt that the disease does not originate *de novo*. In a few days the wound was healed up and nothing more thought of it.

Aug. 11th (53 days later) he was very drowsy and sleepy. On the morning of Aug. 12th, he complained of being sick and was given some purgative medicine, in the mean time, thinking he had made himself sick by going in the creek bathing the day before.

Early in the afternoon he ate a few mouthfuls of melon, which he thought made him sick, and called for salt water to make him vomit, of which he took one swallow and it produced so much disturbance that I was sent for at once. When I entered the room he hailed me, saying, "I made too much wind." Any current of air at this time would produce a spasmodic condition. I found him with a full pulse, 80 per minute; temperature one hundred under the tongue; hands slightly moist, with a cold, clammy feel. Dyspnœa beginning to manifest itself. His tongue had a very white coat on it and he was spitting froth. His mother stated that his tongue had turned white since morning.

I used the following treatment:

| | | |
|---|---|---|
| ℞ | Potassium bromide | ℥ ss. |
| | Aquæ | ℥ iv. |

M. Sig. One teaspoonful every hour.

One dose was given forcibly, and it threw him into a spasmodic condition. He said afterwards he saw a string of stars run through the top of the house and as high as heaven, when he took the medicine, the last thing swollowed.

In the mean time, I had sent for a consulting physician. In the night we gave him morphine and bromidia *per rectum*. It at first had a very sedative effect, but subsequently increased his agony. He complained of no pain, but said he was smothering. He had no dread of moon and daylight, but the light from a lamp would make him scream. During the last nght, the mere opening of a door would make him furious. His cutaneous sensibility was so great that he would suffer from the atmosphere of an open door.

On the morning of the 13th, the day of his death, he became more delirious and furious, wishing all manner of punishment upon his parents, while the night before, he was all the time engaged with them in prayer. The white and tenacious froth continued to increase in his mouth and nose.

He never swallowed anything after I saw him, but this one dose of the bromide solution. He lost his strength by 10 o'clock, and began to vomit a blackish substance, which I supposed to be a decomposition of bile, if not bile in its ordinary condition when vomited from the stomach.

Delirium and hallucinations increased until his death, about two o'clock on the third day of his illness. For the last twelve hours he was in a spasmodic condition, but the spasms were not of a tetanoid character. There were no opisthotonous convulsions, but in his last motion he clenched his jaws, showed his teeth, and drew his head back in an opisthotonous manner.

If your time will permit you to answer, could this case have been anything but hydrophobia?

Does hydrophobia originate spontaneously, and has Pasteur's treatment been a success?

I made no attempt to get the woorara for this case as it is not officinal in this country.

Your Friend and Pupil,
J. D. BRADLEY.

P. S. By the way I used my clinical thermometer under the boy's tongue while he was spitting frothy saliva. Would there be danger in putting it under another's tongue.

